# A Novel Shallow Neural Network-Augmented Pose Estimator Based on Magneto-Inertial Sensors for Reference-Denied Environments

**DOI:** 10.3390/s25226864

**Published:** 2025-11-10

**Authors:** Akos Odry, Peter Sarcevic, Giuseppe Carbone, Peter Odry, Istvan Kecskes

**Affiliations:** 1 Faculty of Engineering, University of Szeged, 6725 Szeged, Hungary; sarcevic@mk.u-szeged.hu; 2 Department of Mechanical, Energy and Management Engineering, University of Calabria, 87036 Cosenza, Italy; giuseppe.carbone@unical.it; 3 Institute of Informatics, University of Dunaujvaros, 2400 Dunaujvaros, Hungary; podry@uniduna.hu (P.O.); kecskesi@uniduna.hu (I.K.)

**Keywords:** gradient descent filter, inertial measurement unit, Kalman filter, pose estimation, sensor fusion, shallow neural network

## Abstract

Magnetic, angular rate, and gravity (MARG) sensor-based inference is the de facto standard for mobile robot pose estimation, yet its sensor limitations necessitate fusion with absolute references. In environments where such references are unavailable, the system must rely solely on the uncertain MARG-based inference, posing significant challenges due to the resulting estimation uncertainties. This paper addresses the challenge of enhancing the accuracy of position/velocity estimations based on the fusion of MARG sensor data with shallow neural network (NN) models. The proposed methodology develops and trains a feasible cascade-forward NN to reliably estimate the true acceleration of dynamical systems. Three types of NNs are developed for acceleration estimation. The effectiveness of each topology is comprehensively evaluated in terms of input combinations of MARG measurements and signal features, number of hidden layers, and number of neurons. The proposed approach also incorporates extended Kalman and gradient descent orientation filters during the training process to further improve estimation effectiveness. Experimental validation is conducted through a case study on position/velocity estimation for a low-cost flying quadcopter. This process utilizes a comprehensive database of random dynamic flight maneuvers captured and processed in an experimental test environment with six degrees of freedom (6DOF), where both raw MARG measurements and ground truth data (three positions and three orientations) of system states are recorded. The proposed approach significantly enhances the accuracy in calculating the rotation matrix-based acceleration vector. The Pearson correlation coefficient reaches 0.88 compared to the reference acceleration, surpassing 0.73 for the baseline method. This enhancement ensures reliable position/velocity estimations even during typical quadcopter maneuvers within 10-s timeframes (flying 50 m), with a position error margin ranging between 2 to 4 m when evaluated across a diverse set of representative quadcopter maneuvers. The findings validate the engineering feasibility and effectiveness of the proposed approach for pose estimation in GPS-denied or landmark-deficient environments, while its application in unknown environments constitutes the main future research direction.

## 1. Introduction

A magnetic, angular rate, and gravity (MARG) sensor unit includes accelerometer, gyroscope, and magnetometer sensors. They have become the de facto standard in current mobile robot systems because they offer characteristics such as low cost, simple interface, and high-resolution measurements. These units are utilized for collecting navigation data such as attitude and heading reference systems (AHRS) to estimate orientation and heading information, as mentioned in [1,2,3,4]. They are also used to perform attitude control and incorporate the results in the simultaneous localization and mapping (SLAM), for example as reported in [5,6,7].

In practice, reference measurements such as GPS signals and visual or landmark information are often not available, for instance due to phase tracking loss and jamming sensitivity. This can be attributed to both the characteristics of the environment and the limitations of the embedded system, which result in serious performance degradation of both the localization algorithms and control approaches that rely on the estimated pose data [8,9]. In these cases, solely MARG-based pose estimation (i.e., 3D positions, velocity, and acceleration vector estimation) becomes crucial and can be seriously affected by the deficiencies of low-cost MARG sensors [10,11,12,13]. The inevitable noise superimposed on the measurements, temperature dependent bias, effect of gravity vector, and magnetic disturbances constitute the main error sources, which make MARG-based pose estimation a difficult problem even when required for only a short period of time [14,15]. Three-dimensional position estimation based on double integration of the acceleration signal provided by a MARG sensor is a challenge even if a robot’s 3D acceleration is known. The aforementioned uncertainties of the measurements become even more significant because of noisy environments, for example as highlighted in [16,17,18].

Pose estimation in mobile robotics is realized within a multi-sensor fusion framework, where relative measurements provide the prediction step of the extended Kalman filter (EKF) and reference measurements (e.g., from LiDAR, camera, or radio beacons) perform the correction step, compensating for drift and other numerical integration errors. Several recent works have successfully demonstrated such approaches: ref. [19], an EKF-based localization method integrating inertial measurement unit (IMU), wheel odometry, and ultra-wideband (UWB) measurements, where a complementary filter processes IMU and odometry data to generate EKF control inputs; ref. [20], an event-triggered EKF framework fusing barometer, IMU, and wheel encoder data for three-dimensional state estimation; ref. [21], a LiDAR-assisted fusion of IMU heading and wheel odometry for indoor localization; ref. [22], a UWB-, odometry-, and AHRS-based Kalman-filter fusion for precise indoor trajectory tracking; ref. [23], a wheel—inertial—visual odometry system using an RGB-D camera, IMU, and encoders enhanced by a fuzzy inference system and an iterated error-state Kalman filter; ref. [24], a multi-sensor fusion factor-graph method combining IMU, odometer, and LiDAR; and ref. [25], a UWB-, IMU-, odometry-, and laser-based fusion employing EKF for robot navigation. These methods represent recent advances that achieve high precision and robustness in robot localization under favorable sensing conditions. However, their reliability depends strongly on the continuous availability of update measurements from absolute sensors (e.g., GPS, camera, LiDAR, or radio beacons). When such reference updates are lost due to environmental or sensor limitations, the system must rely solely on prediction based on relative measurements, leading to rapid degradation of trajectory-tracking accuracy as drift accumulates. In such critical situations, the system’s safety and reliability can only be maintained if accurate motion and acceleration predictions can be derived purely from relative sensors such as MARG units. This inherent limitation of multi-sensor fusion frameworks motivated the present work, which focuses on achieving reliable acceleration vector estimation based exclusively on MARG-only measurements.

Quadrotors are among the largest applications in which reliable pose estimation is an inevitable requirement, as these platforms would otherwise damage both the hardware and its environment [26,27,28]. UAVs are increasingly employed in remote surveillance missions where their flight performance is heavily affected by uncertain environmental changes, external disturbances, and unreliable sensor information. Recent research has addressed these challenges using advanced control and estimation frameworks. Hierarchical control architectures integrating adaptive PID-based MPC have been proposed to improve optimal trajectory tracking under unpredictable environmental conditions [29], while variable disturbance observer-based controllers enhance robustness through adaptive tuning of the Q-filter according to the dynamic model and sensor noise characteristics [30]. High-gain disturbance observers have also been adopted to integrate disturbance estimation with sliding-mode control [31] and higher-order sliding-mode estimators have been utilized to handle parameter variations and perturbations, resulting in improved quadrotor dynamics [32]. More recently, data-driven approaches have emerged in which online learning-based control frameworks fuse uncertainty estimation with nominal controllers to cope with complex and time-varying dynamics [33]. Comparative analyses demonstrate that such hybrid learning-control schemes achieve an advantageous tradeoff between estimation accuracy, real-time performance, and overall flight stability, in particular those using echo state networks. Although robust control frameworks can effectively handle system uncertainties, their performance ultimately depends on the quality of the sensor data. Reliable estimation can only be achieved through accurate processing and fusion of multi-sensor information in order to provide reliable inputs to the control loops. Consequently, recent studies focus on addressing the issue at its source, namely, developing advanced fusion algorithms that can ensure robust state estimation under dynamic conditions.

In [28], the authors reported on an inertial measurement unit-based (IMU-based) pose estimation (which relied on only accelerometer and gyroscope sensors) for quadrotors, where an unscented Kalman Filter (KF) framework was utilized with motor speed- and torque-based unique process models and IMU data driven measurement model. In a similar fashion, [34] derived sophisticated dynamical models for multirotor systems that allow for obtaining accurate state estimation when data updates from reference sensors are unavailable. However, the proposed models require detailed knowledge of the state vector characteristics and noise parameters (e.g., the covariance matrices of KF), and as such are difficult to generalize and practically set up.

Pedestrian dead reckoning is a popular application in which IMU-only algorithms are developed for pose estimation [35,36,37]. In [38], a tightly-coupled EKF framework was proposed for IMU-only pose estimation with the application of neural networks (NN). Significant advantages were demonstrated, which included limited drift errors over other popular state estimation approaches. However, the bandwidth dynamics in pedestrian applications is significantly smaller than that in the case of robots or multirotors. In [39], an IMU-based dead-reckoning algorithm was developed for wheeled vehicles that employed KF and deep NN (DNN) to robustly handle the noise effects in state estimation. A novel state-space motion model was incorporated in the KF framework and deep learning was utilized to dynamically adapt the covariance matrices in the KF, yielding robust performance. Despite the technological advances of small and powerful onboard computers that execute multiple NNs real time, deep learning methods continue to increase computational costs significantly, reducing the applicability of these methods on standard low-cost embedded system-based robot platforms. In the work presented in [40], a triple-channel DNN was developed based on physical and mathematical models of IMUs, resulting in a significant accuracy improvement on the micro-aerial vehicle dataset.

Convolutional NNs (CNN) have also been employed for attitude estimation, for example in [17]. However, similar to DNNs, it is difficult to apply CNN networks within embedded systems because of their large capacity requirements [17]. Similarly, a bidirectional long short-term memory (LSTM) deep learning model was developed for estimating relative 6D poses from noisy low-cost IMU data in [41]. In [42], a complex DNN was employed for velocity estimation based on 9-axis IMU sensor data. Deep networks were successfully applied to estimate vehicle movement directions from accelerometer data [43]. Machine learning methods were used to estimate position, speed, and acceleration from a 9-axis IMU data in an iPhone application with the aim of statistically evaluating the behavior of the driver [44], although with some accuracy limitations.

Given the limitations of the above-mentioned approaches, shallow neural network (SNN) architectures appear to be a promising alternative for addressing the problem. Because the pose estimation problem is characterized by dataset of relatively low input and output dimensions, the processing pipeline is based on individual samples rather than sequences of data; therefore, SNN provides a more suitable topology than DNN. Specifically, SNNs do not introduce unnecessary complexity or require significant memory capabilities, aligning well with the hardware and software requirements of low-cost embedded systems-based architectures and allowing for efficient processing while maintaining adequate performance levels. It was shown in [45] that this approach can significantly reduce computational costs and hardware requirements while providing reliable results when no event recognition is required. The advantage of SNNs their smaller computational cost (similar to an EKF), and consequently their applicability to microcontrollers. Recently, SNNs have also been proven as a feasible solution for the classification of vehicle movements based on accelerometer signals [46]. Cascade-forward NNs (CFNNs) are applicable for time series data [47], especially when the input signal needs to be adjusted with a correction signal. The cascade architecture enables correction of the directly calculated sensor frame acceleration with a mixture of input channels, i.e., magnetic, angular rate and gravity signals, along with estimated orientation angles.

The above comprehensive review reveals that accurate 3D position and velocity estimation based on the integration of acceleration signals remains a challenging task, primarily due to the significant uncertainty of MARG sensor measurements arising from both environmental noise and inherent sensor limitations [16,48,49]. As highlighted in the literature, when reference measurements are unavailable owing to sensory or environmental constraints, the system must rely solely on MARG data to sustain performance, which necessitates the reliable estimation of the true acceleration vector. While sophisticated analytical models can theoretically address this issue, their practical application is hindered by model complexity, high dimensionality, and the presence of unknown dynamics and parameters. Alternatively, data-driven methods such as deep neural networks (DNNs) have shown promising performance, yet their deployment in embedded systems is often infeasible due to excessive computational demands.

Motivated by these challenges, this study aims to identify an optimal SNN topology that balances computational efficiency and estimation accuracy, thereby enabling precise MARG-based acceleration estimation. The proposed approach contributes to achieving consistent velocity and position estimates over short time windows—particularly in the absence of external reference updates—based exclusively on MARG sensor inputs. To this end, the true acceleration is learned using SNNs trained on datasets generated from a validated robot–sensor model, ensuring both practical applicability and generalization capability.

The main contributions of this work are summarized as follows:

1. A complete learning framework is developed that produces a computationally efficient pose estimation model suitable for implementation on microcontroller-based embedded systems. The core element of this framework is an SNN that achieves high estimation performance with low computational cost.
2. A robot–sensor model driven by real-world measurements is constructed to generate the training dataset. This model provides reference data for network training together with realistic MARG sensor readings, forming a comprehensive database for MARG-only pose estimation approaches.
3. A novel CFNN architecture with two hidden layers is proposed. The results demonstrate that this topology represents the most suitable configuration among the SNNs, effectively integrating useful information from various input sensor signals (e.g., raw MARG data and orientation estimates) to learn the complex nonlinear mapping required for estimating the true acceleration.
4. A comprehensive performance evaluation is conducted, including different MARG data configurations within NNs and a comparison with established attitude estimation filters such as EKF-based and gradient-descent algorithms, with the results used to validate the proposed approach.

The remainder of this paper is structured in three main sections: Section 2 introduces the tools and methods applied to develop and evaluate the NN-based algorithms from the real robot setup over the database generation and MARG-based algorithms to the employed NN structure and performance evaluation methods; Section 3 presents the obtained results, analyses, and influence of the parameters on performance; and Section 4 evaluates the results and provides a final overview of both the applicability and performance of the developed methods. Finally, Section 5 concludes the work.

## 2. Proposed Method

**Figure 1:**
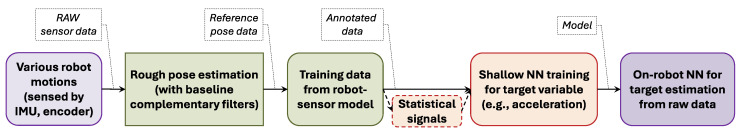
Block diagram of the applied method. The colors represent three main research phases: the rough estimation stage based on robot motions is indicated in green; the training of NNs is highlighted in red; and the real robot activities are indicated in purple.

The main concept of the research is shown in Figure 1. The goal of the trained SNN is to achieve high accuracy in pose estimation. The mobile platform (robot) equipped with multiple sensors (e.g., IMU and encoder) needs to execute various motions; during these motions, the raw sensor data is registered. Then, the proposed procedure applies a rough pose estimation block, where the aim is to approximate both the trajectories and dynamics of executed motions. The output of this stage consists of roughly-estimated 6DOF pose time series, which are supplied to a realistic simulation environment. The simulation environment utilizes a 6DOF dynamical system equipped with realistic MARG sensors, which executes the input trajectories with the desired dynamics. Because the simulated system has an exactly known dynamical model, both the true states (ground truth of spatial coordinates and orientations) of the system and the raw MARG measurements are generated in an annotated database for NN training. This annotated database is also augmented with statistical signals, which represent different features of instantaneous MARG measurements. This enables the obtainment of a comprehensive database composed of multiple realistic robot maneuvers; for each scenario of executed motion, the ground truth data (target), raw sensor measurements (input) and statistical signals (input) are given. These training data are then used to train a shallow NN that estimates the 3D acceleration based on the input signals. This strategy ensures that the trained network shows both better performance and less bias in the estimated pose than the benchmark EKF-based methods during the real robot implementation.

**Figure 2:**
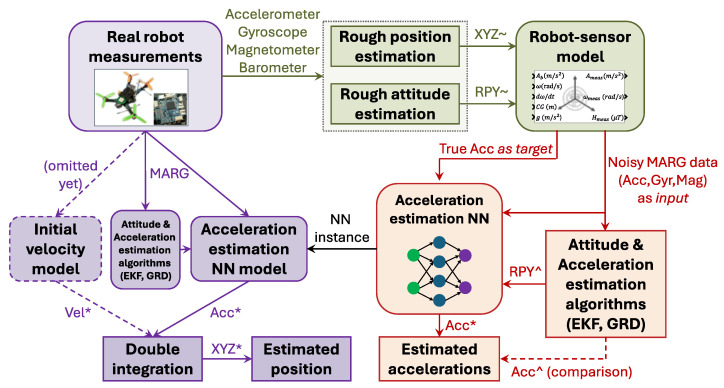
Block diagram of the NN development process. The colors represent three main research phases: the rough estimation stage based on quadcopter flights is indicated in green (estimated position vector is XYZ~, while estimated attitude expressed with roll, pitch, and yaw is RPY~); the training of NNs for acceleration estimation using the generated database is highlighted in red; and the NN implementation process to the robot is indicated in purple. NN-based estimates are indicated with an asterisk in the superscript, whereas EKF- and GRD-based estimates are denoted with a hat symbol in the superscript.

Figure 2 shows the block diagram of the conducted work in more detail. A quadcopter constituted the benchmark setup on which the proposed work was evaluated. The detailed development process is discussed below:

1. First, a physical low-cost quadcopter was used to generate various motions. Multiple trajectories were executed by the robot and the raw sensor data were simultaneously registered on an SD card. This process comprised more than 42 min of quadcopter flight with various maneuvers (i.e., random flights with different acceleration, loop trajectories, flip maneuvers, and dedicated rotations).
2. Next, multiple filtration steps were executed to roughly estimate the pose of the quadcopter during the executed flights. The filtration steps included simple low- and high-pass filters and their combination, with the cut-off frequency adapted to the dynamics of the real system. The applied filters were used to both combine the raw sensor data and remove bias, thereby providing approximated trajectories. The aim of this rough estimation of position and orientation is to enable the generation of real-world scenarios with the proposed simulation model. This method ensured that the NN teaching was based on realistic maneuvers and that the generated signals had the same spectral characteristics as the real-world motions. The measurements included the accelerometer, gyroscope, magnetometer, barometer, and estimated attitude results, from which rough trajectory curves were obtained. These rough trajectories were only approximations (as the real trajectories executed by the quadcopter were unknown); however, the dynamics, shapes, and frequencies included in the obtained trajectories were the same as those in the real flight maneuvers.
3. These roughly estimated pose data were employed within the simulation environment introduced in [49] to generate realistic sensor data along with the ground truth poses; this was a key step, since the ground truth pose was unknown during the real quadcopter flights. Based on the raw sensor data, attitude estimation was executed in two state-of-the-art filter frameworks to obtain the orientation of the quadcopter.
4. After the database was generated, the NN architecture was set up and the NN teaching process was executed. Finally, the obtained NN was evaluated for acceleration, velocity, and pose estimation. The advantage of shallow networks against deep networks lies their simplicity, reduced need for training data, lower susceptibility to overlearning, and easier applicability in embedded systems (with calculation requirements similar to a traditional EKF). The proposed environment executes orientation estimation with EKF and gradient descent (GRD) algorithms, which were used as input signals in the NN teaching process. This partly regularizes the network via physics (similarly to physics-enhanced NNs); moreover, it reduces the required network complexity, since the NN is supplied by compressed and semi-processed data. Additionally, statistical signals (e.g., magnitude) were utilized for similar reasons.

For performance evaluation of these topologies, the Pearson’s correlation coefficient was calculated, which allowed us to estimate the value of a dependent variable regarding a particular value of an independent variable through regression equations. The proposed analysis contributed to setting up the most appropriate NN in terms of real-time pose estimation when sparse reference signals are available.

### 2.1. Accessibility Experiments with a Real Quadcopter System

**Figure 3:**
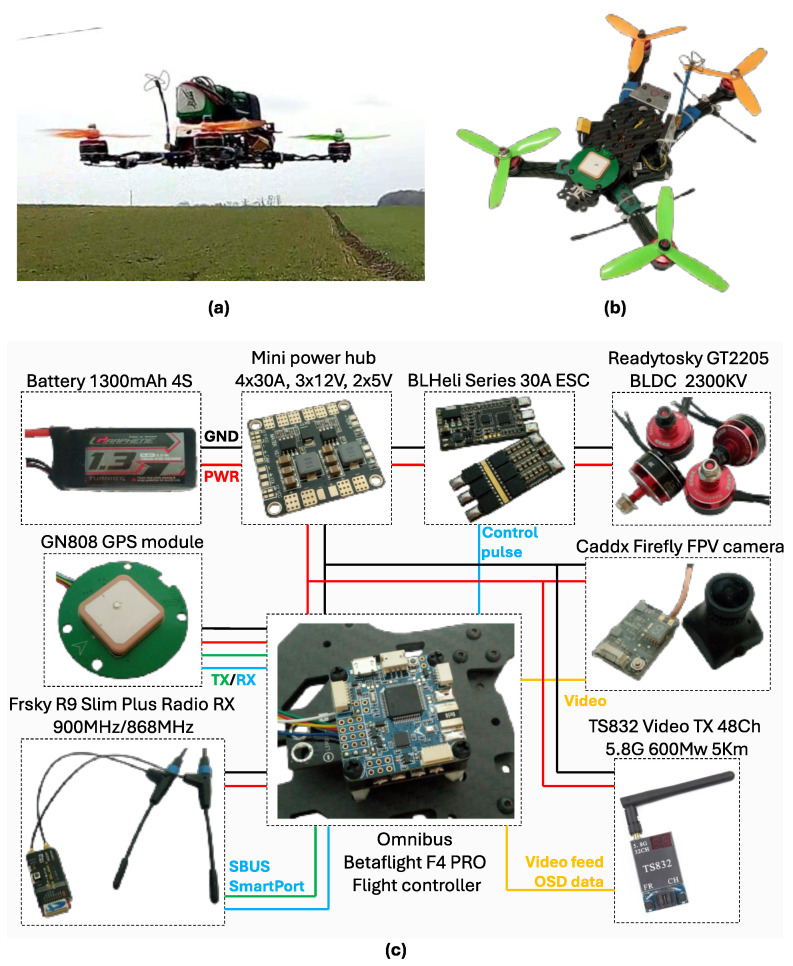
Low-cost quadcopter setup used to generate input signals for database generation: (**a**) quadcopter in flight during measurement, (**b**) the utilized quadcopter setup without the battery, and (**c**) the main components of the quadcopter and their interconnections.

The low-cost quadcopter setup used in this study is depicted in Figure 3. It was constructed from the following modules: an RD290 290 mm Carbon Fiber HexaCopter frame, 5045 Three-Blade Bullnose propellers, Video TX-TS832 600 mW, Radio RX-Frsky R9 Slim Plus, and ESC BLHeli 30A. Further, the quadcopter was equipped with an Omnibus F4 Pro control unit (Airbot Co. Ltd., London, UK), an MPU6050 Accelerometer and Gyroscope IMU platform (TDK InvenSense, San Jose, CA, USA), HMC5883L magnetometer (Honeywell International Inc., USA), and BMP180 Barometer (Bosch Sensortec GmbH, Reutlingen, Germany, see the details in Table 1). All these sensors were mounted on the body of the drone, with the X-axis indicating the front of the drone, the Y-axis representing lateral movement, and the Z-axis denoting the vertical direction. The drone was controlled with a TaranisQX7 controller joystick (FrSky Electronic Co., Ltd., Wuxi, Jiangsu Province, China). During the executed flight maneuvers, measurements were collected though the Betaflight firmware logging module, with MARG signals acquired at a 1000 Hz sampling rate. Various maneuvers were performed by the quadcopter to create a comprehensive and diverse dataset. The generated dataset included typical flight trajectories, attractive rotations and spins, and repeated loops, and is publicly available at [50].

**Figure 4:**
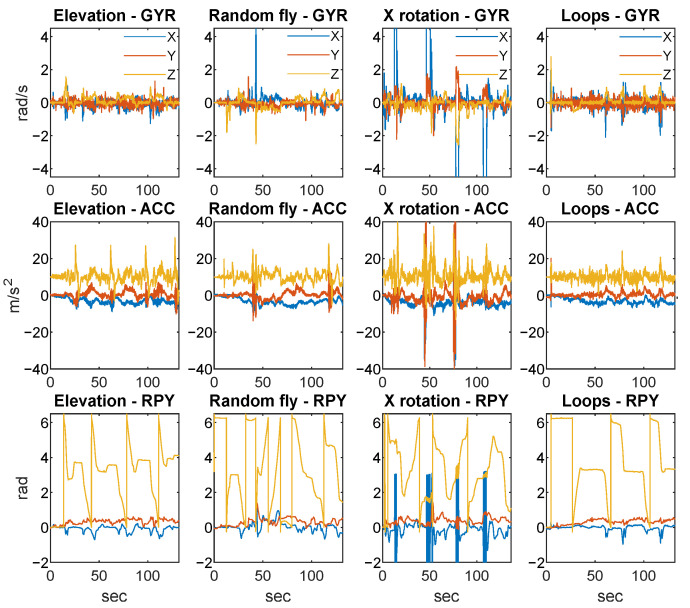
Various scenarios performed by the quadcopter: fast elevation, random flight, rotation around X coordinate, and repeated loops (GYR denotes gyroscope measurements in first row, ACC denotes accelerometer data in the second row, and RPY denotes the calculated attitude in the third row).

Four scenarios are demonstrated in Figure 4: fast elevation, random flight, rotation around X, and repeated loops. These scenarios provided various maneuvers in the training database. The obtained dataset was not suitable for learning from directly due to the lack of ground truth data; for this reason, a simulation model was used to generate the training data, with the real measurements providing the input signals for the simulation.

### 2.2. Rough Estimation of Pose Data

As a first step, rough estimation of pose data was conducted from real quadcopter measurements, with the aim of ensuring similar maneuvers, angles, and frequencies as in reality.

**Figure 5:**
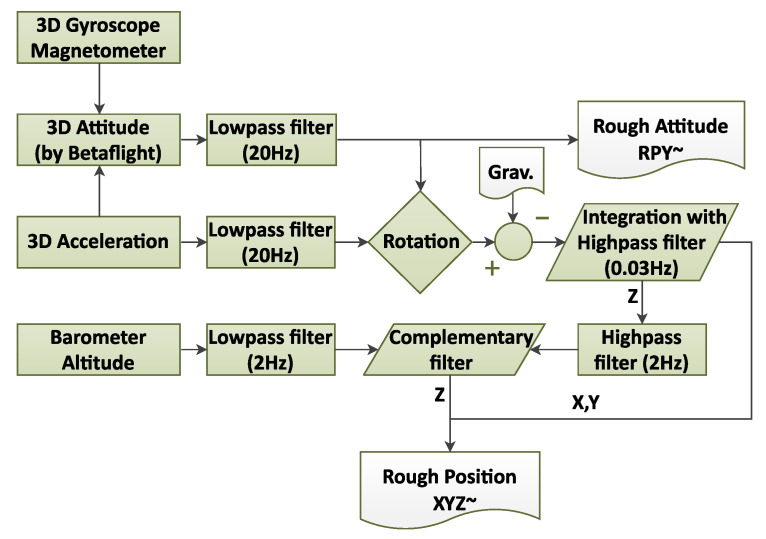
Block diagram of preliminary rough estimation of quadcopter pose (*XYZ~* and *RPY~*).

The Betaflight firmware [51] computes the 3D attitude (roll, pitch, and yaw) using an embedded nonlinear complementary filter [52], which employs a proportional—integral (PI) feedback mechanism to correct gyroscope drift and fuse MARG data for attitude estimation. This filter (depicted on the left side of Figure 5) was used during the initial data collection phase to provide a rough real-time attitude estimate.

Planar coordinates (X, Y) were obtained by integrating the acceleration in the world coordinate system. The acceleration was estimated with a gravity vector decomposition from the accelerometer signals. Double integration was completed by a first-order high-pass filter with a 0.03-Hz cut-off frequency to minimize the drift noise. This cut-off frequency was set empirically, which proved to be an effective compromise between attenuating the low-frequency drift components—mainly originating from accelerometer bias, small orientation errors causing imperfect gravity compensation, and low-frequency sensor disturbaces—and preserving the genuine low-frequency motion dynamics of the quadcopter.

The altitude (Z position) was approximated using another linear complementary filter with a 2-Hz cut-off frequency, wherein the integrated Z position and barometer altitude estimation were fused. Figure 5 shows a block diagram of the rough estimation process.

### 2.3. Simulation Environment

The limitation of the previously introduced quadcopter setup is that the ground truth data (i.e., real positions, velocities, and accelerations) are unknown during the different flights. The flight controller firmware does obtain pose related information of the system; however, these results are based on unreliable estimations with unknown accuracy. Thus, specification of appropriate target signals for the NN was impracticable. To solve this problem, a simulation model described in our earlier work [49] was utilized to generate a database of both true system states and corresponding noisy MARG data based on roughly estimated pose data. The derivation of this simulation model is described in detail in [49]; only key information is provided below.

### 2.4. Model Dynamics

The environment simulates a 6DOF mechanical system that alters the pose of the MARG platform. The system is composed of six joints; these are operated in closed loop with PID controllers. The equations of motion is described in 12-dimensional state space with state vector $x={\left(q,\dot{q}\right)}^{T}$, where *q* represents the vector of the generalized coordinates $q={\left(x_{b},y_{b},z_{b},\phi ,\theta ,\psi \right)}^{T}$ (i.e., MARG pose). The base coordinates of the MARG unit are denoted with $\left(x_{b},y_{b},z_{b}\right)$, whereas the orientation is described with $\left(\phi ,\theta ,\psi \right)$. Motion equations are provided as a closed form as

(1) $$\begin{array}{cc} \dot{x}\left(t\right) & =\left[\begin{array}{c} \dot{q}\\ M{\left(q\right)}^{-1}\left(\tau_{a}-\tau_{f}-V\left(q,\dot{q}\right)\right) \end{array}\right],\\ y(t) & =x(t). \end{array}$$

In Equation (1), M(q) represents the inertia matrix of the system and $V(q,\dot{q})$ includes the potential (gravity) force terms; M(q) and $V(q,\dot{q})$ are derived based on Lagrange-equations, with the variables containing the mass and inertia parameters of each joint (see [49]). Further, $\tau_{a}$ denotes the vector of external forces, whereas $\tau_{f}$ models the friction effects. Each joint is characterized by both static and viscous (damping) frictions. Each joint is operated in the closed loop with PID controllers. The external force of the *i*th joint is defined as (i=1…6)

(2) $$\begin{array}{cc} \tau_{a,i}= & K_{P,i}\left(q_{d,i}-q_{i}\right)+K_{I,i}\int_{0}^{t}\left(q_{d,i}-q_{i}\right)d\xi -K_{D,i}{\dot{q}}_{i}-V\left(q_{i},{\dot{q}}_{i}\right). \end{array}$$

A rational MARG model is implemented at the end of the kinematic chain, which contains scale factor (ΔS) and misalignment (*M*) errors, bias ($\bar{\omega },a_{0},h_{0}$), and white noise components (μ,ν,ϵ) according to Equation (3).

(3) $$\begin{array}{cc} \Omega_{k} & =\left(I+\Delta S_{\Omega }\right)M_{\Omega }\omega_{k}+{\bar{\omega }}_{k}+\mu_{k}\\ A_{k} & =\left(I+\Delta S_{A}\right)M_{A}\left(\alpha_{k}+g_{k}\right)+a_{0}+\nu_{k}\\ H_{k} & =\left(I+\Delta S_{H}\right)M_{H}\left(B_{si}h_{k}+b_{hi}\right)+h_{0}+\epsilon_{k} \end{array}$$

**Figure 6:**
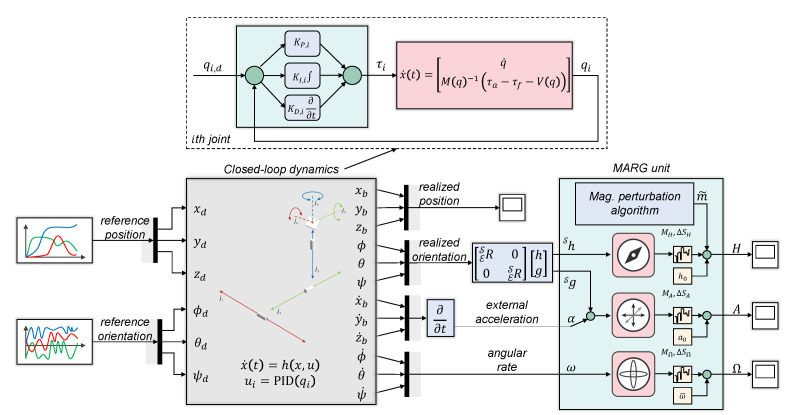
Block diagram of test environment used for the generation of the coherent database of true poses and realistic MARG data.

In the MARG model, Ω denotes the gyroscope measurement, while *A* and *H* represent the accelerometer and magnetometer data, respectively. The 6DOF test bench produces the true states of the MARG (i.e., the real angular velocity ω, real external acceleration α, and real magnetic field vector *h*). Further, an artificial magnetic disturbance generator is employed in the environment to simulate realistic disturbances during the flights. This simulation environment (see Figure 6) was used to generate a database of true system states along with realistic MARG data.

### 2.5. Error Analysis

It is important to note that the aim is to derive the trajectories from real sensor data, despite their lack of precision in terms of derived spatial position signals; the rationale behind this approach is to fill the database with authentic data in order to ensure that the generated trajectories contain realistic acceleration maneuvers akin to those performed by the real drone. The error of the rough estimation stage (Section 2.2) does not significantly impact the performance, since these errors primarily affect the specific trajectories within the dataset rather than the coherence between the ground truth and sensor data in the training and validation databases. Instead, what truly influences the performance of NNs are the underlying characteristics of velocity and acceleration during flight.

**Figure 7:**
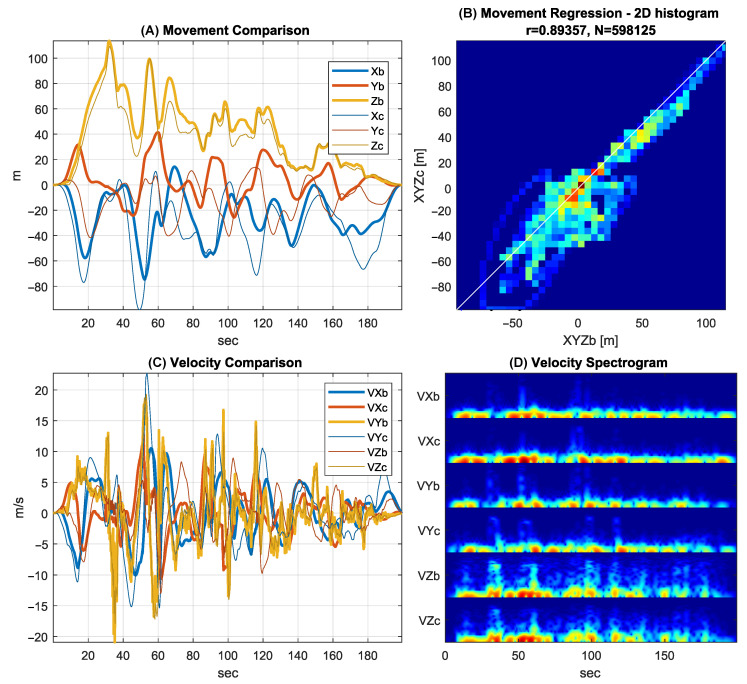
Validation of the model-driven database generation process with error analysis of the rough estimation stage.

Comparative statistics show the error analysis of the rough estimation stage in Figure 7, providing an evaluation of the proposed methodology. This involves taking the modeled sensor measurements (Equations (1)–(3)), running them through the rough estimation algorithm, then comparing the results to those obtained from the estimation algorithm using the original sensor measurements. The difference between these two sets of movement signals represents the error of the rough estimation algorithm while also confirming the characteristic similarities between the real flight movements and the modeled flight movements (i.e., it confirms both the precision and validity of this stage). Figure 7 compares these two set of signals for a flying scenario in which:

(A) The graph shows the estimated position components based on the original sensor data (Xb, Yb, Zb) and modeled sensor data (Xc, Yc, Zc) using the same rough estimation algorithm.
(B) The graph shows the regression analysis of the obtained position signals, where the title contains important metrics such as the Pearson correlation (r = 0.894), root mean square (rms = 18.5 m), and number of used samples (N = 598125).
(C) The graph shows the estimated velocities based on original sensor data (VXb, VYb, VZb) and modeled sensor data (VXc, VYc, VZc) using the same rough estimation algorithm. This represents rather the maneuver characteristics as stated before.
(D) The graph shows the spectrogram of velocity signals, where the rows of spectrogram are listed in the following order: VXb, VXc, VYb, VYc, VZb, VZc. This graph provides a three-dimensional report in which the horizontal axis represents time, the vertical axis represents the frequency, and the amplitude is color-coded. The first two spectrogram rows are the X channels, the third and fourth rows highlight the Y channels, and last two rows show the Z channels. The similarity of these colorful strips confirms the similar spectral characteristics of the pairwise signals.

Figure 7 highlights that the rough estimation stage driven simulation model fulfills its intended role, namely, reliably capturing the characteristics of flight maneuvers. This means that the modeling stage represents the original flight characteristics, allowing the modeling-based database to inherit the realistic flight movement characteristics. As a result, both the robustness and reliability of the proposed method are ensured despite any potential inaccuracies in the rough estimation stage.

### 2.6. Attitude and External Acceleration Estimation

Two popular filter structures were utilized to estimate the orientation based on the noisy measurements.

#### 2.6.1. Extended Kalman Filter

The EKF executes both the quaternion ($q_{k}$) and gyro-bias (${\bar{\omega }}_{k}$) propagation based on gyroscope measurements ($\Omega_{k}$) to obtain the a priori estimate ${\hat{x}}_{k}^{-}={\left({\hat{q}}_{k}^{-},{\hat{\bar{\omega }}}_{k}^{-}\right)}^{T}$. The update phase determines the a posteriori estimate ${\hat{x}}_{k}={\left({\hat{q}}_{k},{\hat{\bar{\omega }}}_{k}\right)}^{T}$ by incorporating the accelerometer and magnetometer measurements zk=AkS,HkST into the model (i.e., measurements in the sensor frame). This is a traditional EKF-based state estimation framework and provides a good balance between estimation accuracy and computational efficiency [53].

The predict phase outputs the a priori state estimate ${\hat{x}}_{k}^{-}$ and the corresponding error covariance $P_{k}^{-}$. The quaternion propagation (${\hat{q}}_{k}^{-}$) is derived by the integration of the rate of change of attitude:

(4) $$\begin{array}{cc} {\hat{x}}_{k}^{-} & =\left[\begin{array}{c} {\hat{q}}_{k}^{-}\\ {\hat{\bar{\omega }}}_{k}^{-} \end{array}\right]=f\left({\hat{x}}_{k-1},u_{k}\right),\;\mathrm{where}\\ {\hat{q}}_{k}^{-} & ={\hat{q}}_{k-1}+\frac{T_{s}}{2}Q\left({\hat{q}}_{k-1}\right)\left[\begin{array}{c} 0\\ \Omega_{k}-{\hat{\bar{\omega }}}_{k-1} \end{array}\right]\;\mathrm{and}\;{\hat{\bar{\omega }}}_{k}^{-}={\hat{\bar{\omega }}}_{k-1}^{-}.\\ P_{k}^{-} & =\Phi P_{k-1}\Phi^{T}+\Sigma_{Q},\;\mathrm{where}\;\Phi =\frac{\partial f}{\partial x}{|}_{{\hat{x}}_{k-1}}. \end{array}$$

The correct phase obtains the a posteriori estimate ${\hat{x}}_{k}$ and the corresponding error covariance $P_{k}$. The predicted observations ${\hat{z}}_{k}^{-}$ are obtained by the quaternion product-based (⊗) coordinate transformations of the reference vectors gE, hE:

(5) z^k−=A^kSH^kS=hx^k−,whereA^kS=q^k−ES⊗gE⊗q^k−*ESandH^kS=q^k−ES⊗hE⊗q^k−*ES.Gk=Pk−ΨTΨPk−ΨT+ΣR−1,whereΨ=∂h∂x|x^k−.x^k=q^kω¯^k=x^k−+Gkzk−z^k−.Pk=I−GkΨPk−.

In Equations (4) and (5), A^Sk denotes the estimated accelerometer measurement in the sensor frame, H^Sk indicates the estimated magnetometer measurement, $T_{s}$ represents the sampling period, $G_{k}$ is the Kalman gain, and the quaternion matrix is denoted with $Q\left({\hat{q}}_{k-1}\right)$. Moreover, $\Sigma_{Q}$ and $\Sigma_{R}$ denote the process and measurement noise covariance matrices, respectively. The derivation of the aforementioned equations is described in detail in our earlier work [54].

#### 2.6.2. Gradient Descent-Based Orientation Filter

This algorithm is characterized by simple structure and shows good performance even in dynamic environments. It incorporates the accelerometer and magnetometer readings within a simple gradient descent algorithm to derive the quaternion derivative, which compensates for the gyroscope measurement error (drift correcting step):

(6) fk=A^Sq^k−AkSH^Sq^k−HkS,∇fk=JkTfk,q^k+1=q^k+Ts12Qq^k0Ωk−β∇fk||fk||,

where β denotes the learning rate of the GRD algorithm and $J_{k}$ denotes the Jacobian matrix of the objective function $f_{k}$ [55]. The gain of the filter is obtained based on earlier findings [49].

#### 2.6.3. External Acceleration Estimation

External acceleration was estimated based on the obtained orientation results with the EFK or GRD algorithm, i.e., as a difference between the gravity and raw measurement vectors:

(7) α^k=Rq^kSEAkS−gE

where gE=(0,0,9.8)T denotes the constant reference vector in the inertial frame and RES∈SO3 indicates the rotation matrix, which is constructed with the estimated quaternion.

### 2.7. Neural Network Model

Each training task requires a specific network architecture and algorithm parameterization [56]. In this research, a minimally sized shallow network was sought that could effectively process the sensor data by orientation sample-by-sample.

Cascade-forward NNs (CFNNs) are similar to feed-forward networks, but include a connection from the input and every previous layer to following layers. The cascade topology of the network supports the “correction signal” logic, which has a good fit to the knowledge-based algorithms. In this work, we compare one hidden layer with a larger number of neurons (CFNN1) and two hidden layers with a smaller number of neurons (CFNN2).

Time-delay NNs (FTDNNs) are regularly used for time series signal processing [57], and have similar memory-like behavior as recurrent NNs. Time series data often exhibit temporal dependencies, with the current value depending on past values. By including delayed-input samples, the NN can capture these dependencies. This allows the model to consider the history of the time series, which is crucial for making accurate predictions. We also used this network for trial and comparison.

**Figure 8:**
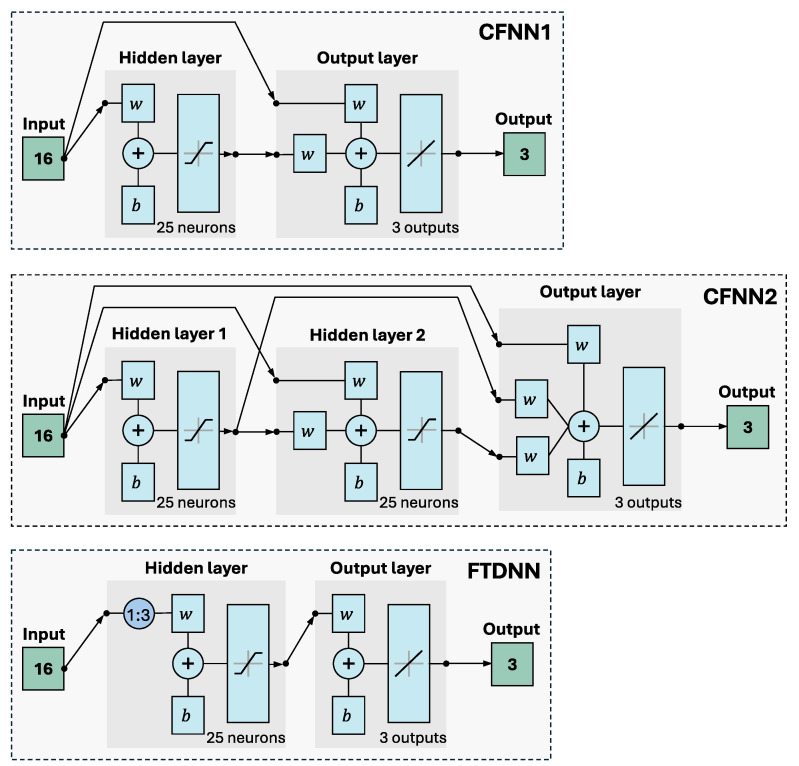
Block diagram of both CFNNs (with one and two hidden layers) and FTDNN with 16 input, 25 hidden, and three output neurons; the structures were generated using MATLAB R2024b.

Therefore, this research tested the following three types of NNs for accurate 3D acceleration estimation (see Figure 8):

1. CFNN1—Cascade-forward NN with one hidden layer, which provides lower nonlinear capabilities. No time delay.
2. CFNN2—Cascade-forward NN with two hidden layers, with the aim of providing higher nonlinear capabilities. No time delay.
3. FTDNN—Focused time-delay NN with three time delays (1:3) and one hidden layer; for example, this network artificially adds an extra 48 input neurons for the 16 real input channels, which results in 64 neurons altogether.

Each developed shallow NN constitutes a regression model with 3–16 dimension inputs and three-dimensional output. Table 2 provides a network structure comparison that lists the key parameters of the addressed architectures. Table 3 summarizes both the characteristics of the utilized database and the inputs and outputs of the addressed problem.

### 2.8. Dataset Splitting and Performance Evaluation

The original measurements were sampled at 1000 Hz; however, downsampling to 100 Hz was employed because the position estimation stage was executed at lower frequencies. This sampling rate was sufficient for both tracking and controlling dynamical systems. When joining all the available scenarios this made for length measurements of 2500 s, for 250,000 samples, which were split into three defined sets where scenarios were ordered randomly: training (TR, 70%, 175,000 samples), validation (VA, 15%, 37,500 samples), and test (TE, 15%, 37,500 samples). The samples were used in the original order because of the previous samples in FTDNN, and repeated at the beginning as needed.

The performance of the velocity or acceleration estimation can be represented by the Pearson’s correlation coefficient, as in [58]. A value >0.9 is interpreted as good accuracy, since the baseline filter methods reach only 0.5–0.7 (see Table 4). The estimation performance in this research was calculated using the Pearson correlation between the ground truth and estimated accelerations, which was applied for the three directions (X, Y, Z) and for the overall samples A:

(8) $$r\left(a\right)=\frac{\sum \left(a_{i}-\bar{a}\right)\left(b_{i}-\bar{b}\right)}{\sqrt{\sum {\left(a_{i}-\bar{a}\right)}^{2}\sum {\left(b_{i}-\bar{b}\right)}^{2}}},$$

where *i*, *b*, and *a* represent the sample iterator, reference acceleration with its average value $\bar{b}$, and estimated acceleration with its average value $\bar{a}$, respectively.

The aforementioned approach provides unitless comparison metrics. The correlation is calculated separately for the X, Y, and Z axis channels and for all three axes at the same time (denoted with A), which represents the overall performance. The three axes are joined with simple concatenation A = [X; Y; Z]. It is possible to calculate the following four performances for the three sets of data, i.e., training: rTR(X), rTR(Y), rTR(Z), rTR(A); validation: rVA(X), rVA(Y), rVA(Z), rVA(A); and test: rTE(X), rTE(Y), rTE(Z,) rTE(A), from which the independent test set performance is used for the evaluation of rTE(A).

The observed overfitting was marginal because of the small number of neurons in the network compared to the large number of training samples. The training correlation performance was slightly higher than the test performance; however, this was a very small difference, e.g., rTR(A)-rTE(A) ≈ 0.005 for the largest NN that was trained. Consequently, the overfitting can be considered negligible for these shallow networks.

### 2.9. Network Initialization and Training Method

Levenberg–Marquardt backpropagation was used for the aforementioned SNNs because it provides better performance compared to gradient training. The GPU could not be utilized for faster training, since the GPU calculations only support gradient training and not Jacobian training. The Levenberg–Marquardt backpropagation algorithm uses the Hessian matrix as the quasi-Newton methods, and its mathematical description can be found in the official documentation [59].

The weights of NNs must be initialized to small random numbers because of the stochastic optimization algorithm [60]. Owing to both this stochastic nature and randomness in the initialization, the same network trained on the same data can produce different results. Validating using only one network can provide a rough performance estimation, because there are performance variances between differently initialized networks; however, in this paper, only one network is calculated for each configuration due to the long calculation time. CPU-based training requires 3–24 h per network (on a desktop PC with Intel-i5 and 16 Gb RAM configuration). We accept this performance estimation because its variation was only var(r) ≈ 0.003, verified by 30 Monte Carlo repeating random samples and random network initialization.

Because the embeddability of the target NN is highly dependent on its computation resource, it is important to choose the proper network size in such a way that it reduces the number of neurons as much as possible while keeping performance as high as possible.

The executed training process was based on the default hyperparameters provided by MATLAB’s *cascadeforwardnet* function for several reasons as follows.

1. Dataset size: Our dataset was sufficiently large, which often mitigates the need for extensive hyperparameter tuning. With a large dataset, NNs tend to exhibit robustness to variations in hyperparameters.
2. Convergence: We observed satisfactory convergence of the NN training process with the default parameters. This convergence indicates that the default parameters were suitable for our dataset and task.
3. Low variability: The variability in the performance of the NN outputs was minimal, indicating that the default parameters provided both stable and consistent results across different runs.

The utilized hyperparameters are provided as follows.

- Learning rate: 0.01
- Activation function of hidden layers: logistic sigmoid
- Activation function of output layer: linear activation function
- Training algorithm: Levenberg–Marquardt backpropagation
- Mini-batch size: entire training dataset
- Performance function: mean squared error (MSE)
- Regularization: no regularization
- Normalization: no normalization
- Stop criteria
  - Maximum number of epochs: 1000
  - Maximum time: infinity (no limit)
  - Performance goal: 0 (perfect case)
  - Maximum gradient: $1\times 10^{-7}$
  - Damping parameter (μ): 0.001
  - Training stops: if $\mu >1\times 10^{10}$
  - Maximum validation checks: 6

## 3. Results

The final solution was selected after three performance-based evaluation: first, the combination of input channels; second, the help of statistical channels; and third, the optimal number of neurons. A network with fast training characteristics and a single hidden layer was used in the first benchmark (CFNN1), whereas a network with two hidden layers was employed in the second benchmark (CFNN2). Finally, the larger networks were run with more neurons. Keeping this order of training helped to shorten the whole training procedure.

The first set of comparisons constituted both the sensor data and orientation filter outputs (generated by the EKF and GRD algorithms). The second set then incorporated additional statistical channels in the performance evaluation process, e.g., magnitudes of the accelerometer and gyroscope signals.

The third comparison investigated the optimal size of the networks. The selected final best model consisted of a CFNN2 architecture with H = 34 hidden neurons (network CFNN2H34), and the development leading to this solution is presented in what follows.

### 3.1. First Performance Comparison—Raw Sensor Data and Orientation Angles

Table 4 lists the obtained performance results, which include both the reference baseline methods and the NN models used for comparison. All performance results are expressed with correlation indexes between the ground truth acceleration and estimated acceleration. Table 4 lists three groups of results, which are discussed as follows.

1. The reference baseline group shows the baseline method estimation results without NN elaboration. In the evaluated cases, the orientation is estimated with the EKF or GRD method based on the accelerometer, gyroscope, and magnetometer measurements (Acc + Gyr + Mag).
2. The applicable NN group represents the elaborated solutions that can be applied in real mobile robot systems. The input fully relies on the accelerometer, gyroscope, and magnetometer measurements (Acc + Gyr+ Mag).
3. The reference NN group uses the ground truth orientation instead of the estimated orientation (ANG), which proves the theoretical maximum performance in the case of a perfect attitude estimation.

The best results are marked in bold for each group. The abbreviations are as follows: Acc, Gyr, Mag, EKFe, EKFq, GRDe, GRDq, ANGe, and ANGq represent the accelerometer, gyroscope, magnetometer, orientation estimation with EKF in Euler representation, orientation estimation with EKF in quaternion representation, orientation estimation with GRD in Euler representation, orientation estimation with GRD in quaternion representation, ground-truth orientation in Euler representation, and ground-truth orientation in the quaternion representation, respectively. The acceleration results generated by already-existing methods (i.e., EKFa generated by EKF and GRDa generated by GRD) are included; for example, in Table 4, AccGyrMagEKFeEKFa indicates that 3D acceleration EKFa results are included along with the estimated 3D Euler representation EKFe. The abbreviation of the NNs comprises two parts: NN topology and number of hidden neurons; e.g., CFNN1H18 means a cascade-forward network with one hidden layer (CFNN1) and 18 neurons per hidden layer (H18).

Based on the achieved results, the performance evaluation is discussed as follows. The EKF method yielded the highest correlation results from the baseline groups (r = 0.725). The best reference NN was the AccGyrANGe version (r = 0.964); however, the version with an additional magnetometer was very similar (AccGyrMagANGe). These results prove that the most important factor is the correctness of the estimated attitude angles for the NN-based acceleration estimation, as the reference NNs using the ground truth attitude reached fairly high correlation results. The best applicable NN was the AccGyrMagGRDe combination (r = 0.848), which was halfway between the baseline and theoretical solution. The quaternion or Euler format of orientation only slightly influenced the performance. The contribution of output (GRDa, EKFa) of the baseline method in NN did not increase the performance.

The input channel importance test indicated that NNs mostly relied on the accelerometer and magnetometer channels (see details in Appendix B).

### 3.2. Second Performance Comparison—Additional Features

In the second benchmark, an NN architecture with higher nonlinearity was used. The CFNN2H15 network was characterized by two hidden layers, and each hidden layer had 15 neurons. The required training time of this network was five times higher than the CFNN1H18 network used in the first comparison. As an additional input, some simple feature channels were included, namely, IMUf included four features calculated on accelerometer and gyroscope signals (see Table 5). The last two features are average values on the last 20 samples, and represent the last 200 ms average signal characteristics. This time window was defined empirically.

Table 6 summarizes the obtained performance results. The first row highlights the best applicable NN solution from the previous benchmark (Table 4), which is included in the table for comparison. Two reference NNs were evaluated to determine how the true angle influences the network performance.

The best applicable NN was driven by the AccGyrMagGRDeIMUf input combination. This NN received 16 channels: three accelerometer inputs (AX, AY, AZ), three gyroscope inputs (GX, GY, GZ), three magnetometer inputs (MX, MY, MZ), three estimated angles based on the gradient method (GrdX, GrdY, GrdZ), and foue feature channels (ACCmag, GYRmag, ACCma20, ACCdma20). This NN architecture is evaluated in different tests in the next subsection and in two additional tests in both Appendices Appendix A and Appendix B.

### 3.3. Third Performance Comparison—Number of Neurons

**Figure 9:**
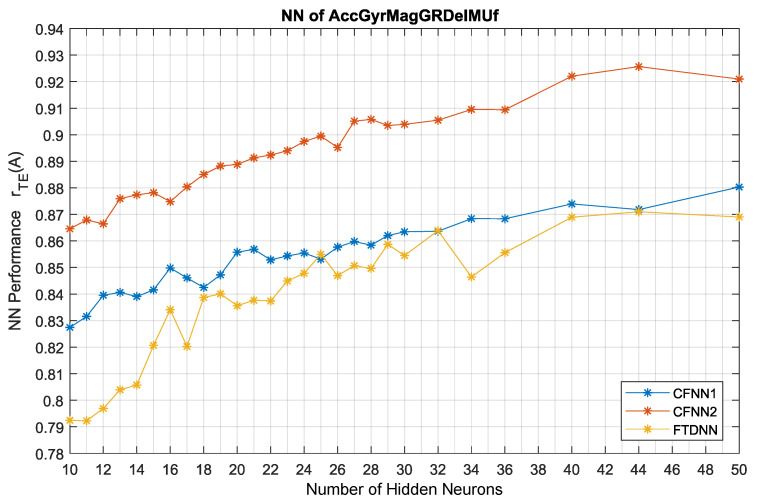
NN performance comparison based on the number of hidden neurons.

Three types of NNs were evaluated in a comparison to analyze the number of hidden neurons; the results are highlighted in Figure 9. The network had 16 input channels; therefore, the optimal applicable number of neurons was estimated as twice the number of input channels, i.e., 32 neurons. The overfitting was measured as the performance difference between the training, validation, and test sets of samples. The overfitting rate in the case of the maximum number of neurons (H50) was only Δr = 0.005, which is similar to the measurement error expressed from performance variability owing to the NN initialization. The NN configuration above 50 neurons (50 + 50 neurons in case of CFNN2) would have high processing requirements for memory to perform calculations in a real embedded implementation. Therefore, NNs with >50 neurons were not evaluated in this comparison; even >35 neurons per layer is not considered applicable in embedding, and would require higher resource compared to an EKF. In the case of CFNN2, there were two hidden layers; therefore, this network had double the number of neurons. This is the principal reason why it had higher performance compared to the CFNN1 version with only a single hidden layer. With 32 neurons, CFNN1 reached the performance level of CFNN2 with 10 + 10 neurons. This indicates the advantage of a double hidden layer over a single hidden layer, even one with twice the number of neurons. The maximum performance was reached when CFNN2 had 45 neurons; however, the version with 34 neurons was selected due to its having similarly high performance. Based on these results, the CFNN2H34 configuration is proposed for subsequent applications.

The FTDNN starts with lower performance; however, above 24 neurons it yields similar performance results to CFNN1. Moreover, FTDNN shows higher performance variability (nonlinearity), which suggests that its performance is more dependent on the weight initialization (in other words, the training algorithm is not so effective). We conclude that the FTDNN-type NN is not worth using for acceleration estimation purposes compared to CFNN type networks.

## 4. Discussion

**Figure 10:**
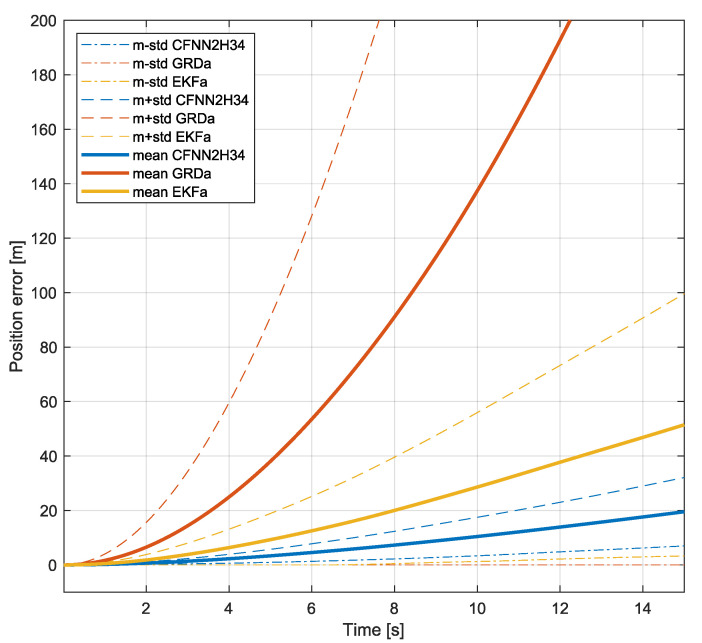
Median position error (Euclidean distance) over time for the proposed CFNN2H34 method and the two baseline algorithms (GRDa and EKFa).

The accuracy of MARG-based 3D position estimation is influenced by the errors of both the NN-based estimated acceleration and of the double integration method. The proposed NN-based acceleration estimation allows the 3D position of moving objects to be derived for short time windows, i.e., estimation results with a length of 5–10 s are characterized as reliable outputs. Table 7 shows typical 3D position errors after 5, 10, and 15 s for double integrated acceleration signals obtained based on the GRD method (GRDa), EKF method (EKFa), and the developed shallow network (CFNN2H34). The typical values are expressed with the median value of 100 sample periods taken from the validation test set. While each method shows high position errors, the proposed NN-based method is characterized by significantly smaller errors. These errors are 0.5–2 m after 5 s, 2–4 m after 10 s, and 5–10 m after 15 s. Figure 10 supports these results by highlighting the median position error (Euclidean distance) as it grows over time (0–15 s) for the proposed method (CFNN2H34) and the two baseline methods (GRDa, EKFa). This provides a more intuitive visualization of the performance improvement.

**Figure 11:**
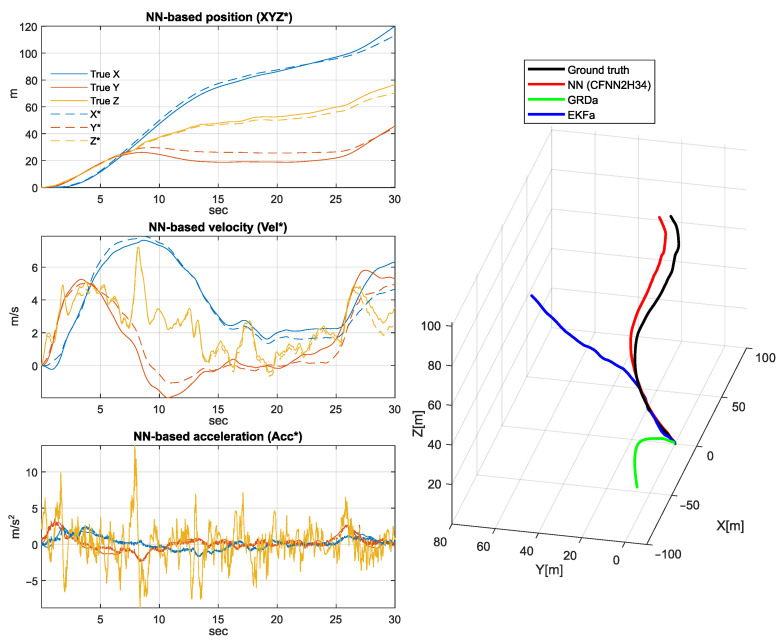
Three-dimensional position, velocity, and acceleration estimation based on the obtained NN model. The acceleration was estimated by the best applicable NN (CFNN2H34) on AccGyrMagGRDeIMUf.

**Figure 12:**
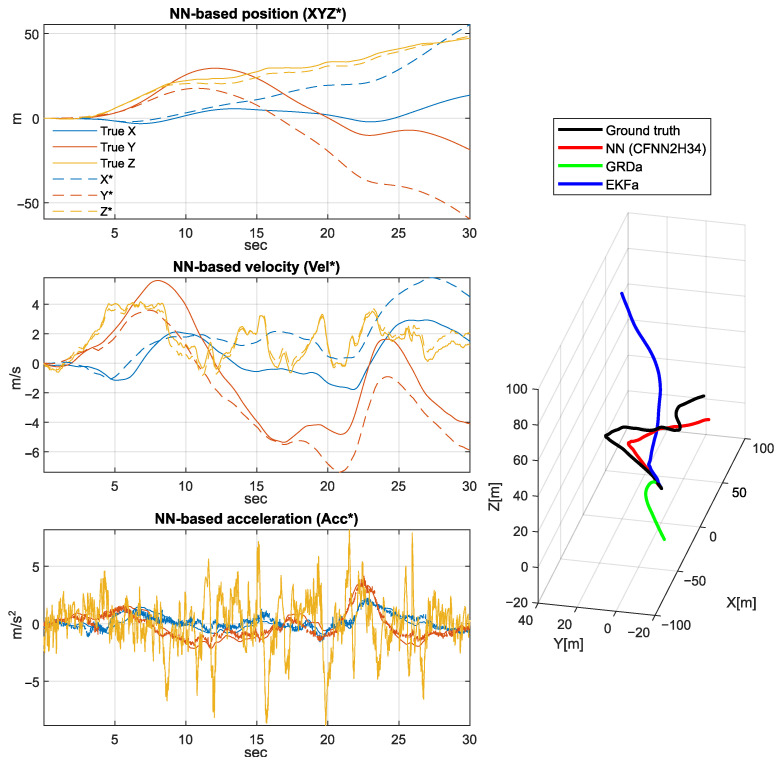
Another example of 3D position, velocity, and acceleration estimation based on the obtained NN model (second scenario).

**Figure 13:**
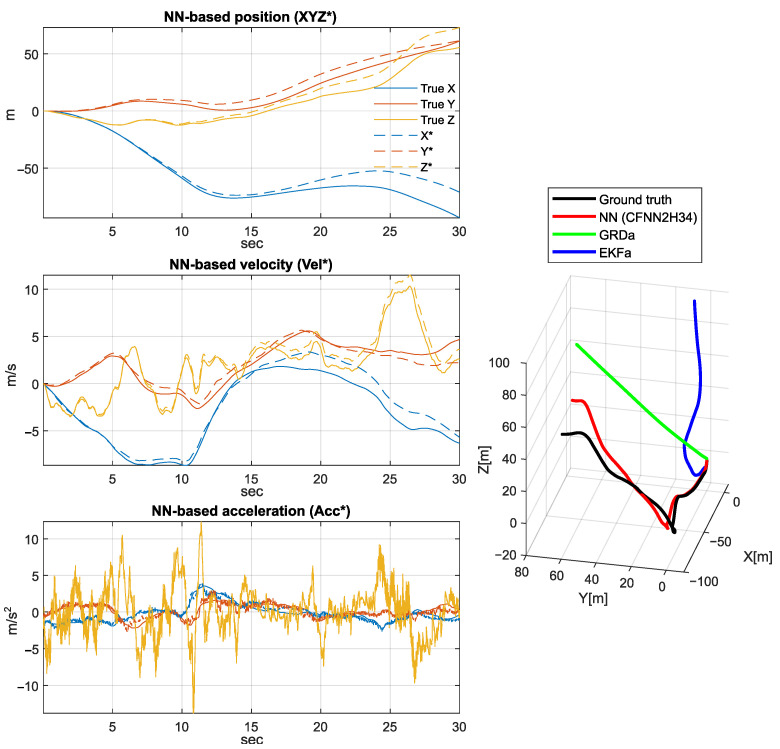
Third scenario of 3D position, velocity, and acceleration estimation based on the obtained NN model.

Figure 11, Figure 12 and Figure 13 depict a few scenarios of the estimated quadcopter movements obtained by simple double integration of the estimated acceleration. The figure plots 30-s integration results, with samples taken from the validation set. The accumulated drift error generates misleading results very soon; however, the proposed NN shows the most similar trajectory shape compared to the ground truth.

The left side of Figure 11, Figure 12 and Figure 13 highlights the NN-based position (XYZ*), velocity (Vel*), and acceleration (Acc*) signal coordinates compared to the ground truth coordinates, while the right side shows the executed spatial trajectory with the black curve (i.e., the ground truth). In addition, the red curve indicates the 3D position derived by the NN-based acceleration estimation, the green curve highlights the 3D position obtained based on the output of GRD algorithm, and the blue curve indicates the 3D position derived based on the Extended Kalman filter results.

The runtime of the proposed NN was compared to the EKF runtime using same PC and same samples. Despite MATLAB implementations of these algorithms not exactly representing the run-time in an embedded system, this provides a rough estimation of the expected processing resources. The analysis shows that CFNN2H34 has a smaller runtime compared to EKF with 20% based on 1000 samples. This means that the calculation resource of the EKF is similar to run a smaller shallow NN. The proposed CFNN2H34 requires 5449 floating point operations (FLOPs) for one input sample (including addition, multiplication, division, and exponential). This is a very low computational load that is well within the capabilities of typical low-cost microcontrollers (e.g., ARM Cortex-M4/M7), which are rated for millions of FLOPs per second (MFLOPs). Additionally, the network has a total of 2567 trainable parameters (weights and biases). Storing these as 32-bit floating-point numbers requires approximately 10.3 kB, which is a minimal footprint for modern microcontrollers. The RAM memory required for storing neuron activations during a forward pass is negligible, on the order of a few hundred bytes. The proposed model is extremely lightweight, orders of magnitude smaller than even simple DNNs such as LeNet-5 (≈60 k parameters, 1–10 MFLOPs) or small LSTM networks (≈50–200 k parameters, 1–5 MFLOPs). These low requirements for both computation and memory strongly support the feasibility of implementing the proposed model on low-cost embedded hardware.

The aforementioned results show that the proposed NN-based 3D tracking algorithm provides useful information of the movement of dynamical systems such as quadcopters. The algorithm is useful when the localization of the moving object should be obtained solely based on MARG data because of the limitation of the environment; for instance, GPS signals are commonly characterized by phase tracking loss, jamming sensitivity, and a discontinuous nature. In these cases, appropriate robot poses should be supplied for the path planning and control algorithms, otherwise the system may damage its environment. The proposed method provides significantly better estimates than the benchmark approaches, which is clearly presented by Figure 11, Figure 12 and Figure 13 as well as Table 7. This method can be improved in the future with more sophisticated integration methods and incorporation of additional sensor signals (i.e., barometer signals are very useful in the altitude estimation). Similarly, evaluation of the impact of hyperparameters on the calculation results could contribute to the comprehensive understanding and optimization of the proposed NN models. These issues are left open for future studies. Although the presented network was trained on data from a single quadcopter platform, thereby capturing platform-specific dynamics and sensor noise characteristics, the proposed methodology remains generic and can be straightforwardly applied to other systems through retraining with platform-specific datasets.

## 5. Conclusions

This paper proposes a new procedure for improved 3D pose estimation based on MARG data fusion in combination with an SNN. The proposed approach is based on four main stages: data acquisition on a real dynamical system, rough pose estimation with baseline filtering, application of both model dynamics and sensor models for database generation for NN teaching, and an optimal NN architecture suited for use in microcontroller-based systems and developed in combination with an orientation filter (EKF or GRD) for improved pose estimation. A specific case study has been investigated by reference to a quadcopter scenario. In particular, a database of real quadcopter maneuvers containing both realistic maneuvers and magnetic disturbances was generated by an appropriate simulation environment to successfully train NNs without significant overlearning. A comprehensive evaluation of NN setups was conducted, including the impact of raw sensor inputs, derived signals and signal features, sampling rate, channel importance, number of hidden layers, and number of neurons.

It was found that a CFNN with two hidden layers and 2×34 neurons was the optimal topology for accurate MARG-only acceleration estimation. The results showed that this NN can reach a significantly higher performance compared to the state-of-the-art methods (EKF or GRD). The Pearson correlation between the reference and estimated acceleration for the presented shallow NN was 0.878, whereas in the case of EKF and GRD we obtained results of 0.725 and 0.491, respectively. The performance of the proposed NN increased with more hidden neurons; however, as its implementation was also considered, we set 35 neurons set as the maximum number per layer in the investigation process. The acceleration vector provided by the obtained topology yielded significantly more reliable position/velocity estimates than the baseline methods. Based on the performed quadcopter flights, the NN-based approach was characterized by a position estimation accuracy with an error range of 0.6–1.5 m in a 5 s time window and 1.9–4.2 m in a 10 s time window.

As supervised methods, shallow NNs represent powerful models for both aggregating available measurements and estimating the external accelerations of mobile platforms. Augmenting an orientation filter with an SNN results in a MARG-only pose estimator that provides reliable position/velocity estimates for shorter time windows in reference-denied environments. Future work will include validation of the proposed method during quadcopter maneuvers, where GPS measurements will be used to generate the reference trajectories. In addition, the integration of invariant filters (e.g., invariant EKF) will be investigated in order to improve robustness and accuracy in dynamic maneuvers.

## Figures and Tables

**Table 1:** Specifications of the sensors used in the measurement setup.

Sensor	Type	Key Parameter	Range	Resolution/Noise
MPU6050	3-Axis Gyroscope	Angular Velocity	User-programmable: ±250 deg/s to ±2000 deg/s	16-bit ADC Noise: ∼0.005 deg/s/$\sqrt{Hz}$
	3-Axis Accelerometer	Acceleration	User-programmable: ±2 g to ±16 g	16-bit ADC
HMC5883L	3-Axis Magnetometer	Magnetic Field	±8 Gauss (full-scale)	12-bit ADC Field resolution: 2 milli-Gauss Noise Floor: 2 milli-Gauss
BMP180	Barometric pressure, temperature, and altitude sensor	Pressure	300 to 1100 hPa (+9000 m to −500 m relative to sea level)	16 to 19-bit output Noise (High-Res Mode): 0.02 hPa (∼0.17 m)

**Table 2:** Key structural parameters of the evaluated neural network architectures.

Architecture	Input Dimension (Real Channels)	No. of Hidden Layers	Neurons per Hidden Layer	Total Hidden Neurons	Time Delay
CFNN1	16	1	H (variable, 10–50 tested)	H	No
CFNN2	16	2	H (variable, 10–50 tested)	2 × H	No
FTDNN	16	1	H (variable, 10–50 tested)	H	Yes (1:3 delays)

**Table 3:** Overview of the database channels and their characteristics.

Source	Channel	Symbol	Unit	Fs (Hz)	Role
Sensors on quadcopter and Beta flight calculations	3D accelerometer sensor signal	ACCb	m/s_2_	1000	Input measurements for database generation based on the 6-DOF test environment
	3D gyroscope sensor signal	GYRb	deg/s	1000	
	3D magnetometer sensor signal	MAGb	uT	1000	
	Barometer (altitude) sensor signal	BARb	m	1000	
	3D attitude (orientation)	RPYb	rad	1000	
Rough estimation of quadcopter pose	Rought 3D position	XYZ∼	m	1000	
	Rought 3D attitude (orientation)	RPY∼	rad	1000	
Output of 6-DOF test environment	Reference 3D acceleration	True Acc (Ground truth)	m/s_2_	1000	Target for NN training
	Reference 3D position	True XYZ (Ground truth)	m	1000	3D position validation
	Noisy 3D accelerometer sensor signal	Acc	m/s_2_	1000	NN inputs
	Noisy 3D gyroscope sensor signal	Gyr	deg/s	1000	
	Noisy 3D magnetometer sensor signal	Mag	uT	1000	
Filter algorithms	Orientation estimation by EKF	EKFe, EKFq	rad	100	NN inputs (RPY^ in Figure 2)
	Orientation estimation by GRD	GRDe, GRDq	rad	100	
	3D acceleration estimation based on EKF orientation results	EKFa	m/s_2_	100	Baseline methods (Acc^ in Figure 2)
	3D acceleration estimation based on GRD orientation results	GRDa	m/s_2_	100	
Additional features (IMUf)	Magnitude of 3D accelerometer signal	ACCmag	m/s_2_	100	NN inputs
	Magnitude of 3D gyroscope signal	GYRmag	deg/s	100	
	Moving average of accelerometer signal applied on the last 20 samples	ACCma20	m/s_2_	100	
	Moving average of accelerometer derivatives applied on the last 20 samples	ACCdma20	m/s_2_	100	
NN output and derived signals	Estimated 3D acceleration	Acc*	m/s_2_	100	NN Output
	Estimated 3D velocity	Vel*	m/s	100	Derived output
	Estimated 3D position	XYZ*	m	100	Validation output

**Table 4:** First performance comparison of NNs on different inputs.

Group	Model Parameters		Performance (Correlation)			
	**Model and Channels**	**Input Dim**	**X**	**Y**	**Z**	**All**
Reference—baseline	Sensor acceleration (Acc)	3	0.422	0.205	0.891	0.265
	GRD on AccGyrMag (GRDa)	9	0.178	0.354	0.782	0.491
	**EKF on AccGyrMag (EKFa)**	9	**0.548**	**0.479**	**0.933**	**0.725**
Applicable NN (CFNN1H18)	NN on Acc	3	0.538	0.210	0.933	0.674
	NN on AccGyr	6	0.570	0.565	0.940	0.758
	NN on AccMag	6	0.709	0.611	0.946	0.803
	NN on AccEKFe	6	0.676	0.562	0.943	0.783
	NN on AccEKFq	7	0.650	0.575	0.941	0.779
	NN on AccGRDe	6	0.573	0.412	0.935	0.721
	NN on AccGRDq	7	0.567	0.414	0.933	0.717
	NN on AccGyrEKFe	9	0.697	0.639	0.947	0.808
	NN on AccGyrGRDe	9	0.593	0.602	0.941	0.772
	NN on AccGyrMag	9	0.728	0.703	0.946	0.830
	NN on AccGyrMagEKFe	12	0.751	0.702	0.948	0.837
	**NN on AccGyrMagGRDe**	12	**0.779**	0.728	0.945	**0.848**
	NN on AccGyrMagEKFeEKFa	15	0.745	0.722	**0.949**	0.841
	NN on AccGyrMagEKFeGRDa	15	0.766	**0.730**	**0.949**	**0.848**
	NN on AccGyrMagGRDeGRDa	15	0.762	0.729	0.946	0.845
	NN on AccGyrMagGRDeEKFa	15	0.778	0.722	0.948	**0.848**
Reference NN (CFNN1H18) with ground truth angles	NN on AccANGe	6	0.969	0.955	0.959	0.957
	**NN on AccGyrANGe**	9	**0.977**	**0.973**	**0.960**	**0.964**
	NN on AccGyrANGq	9	0.959	0.956	**0.960**	0.956
	NN on AccGyrMagANGe	12	**0.977**	**0.973**	**0.960**	**0.964**

*Bold numbers indicate the best results within each group.*

**Table 5:** The additional feature channels (IMUf).

Name [Unit]	Description	Equation
ACCmag [m/s_2_]	Magnitude of 3D accelerometer signal	$ACCmag_{i}=\sqrt{AX_{i}^{2}+AY_{i}^{2}+AZ_{i}^{2}}$
GYRmag [deg/s]	Magnitude of 3D gyroscope signal	$GYRmag_{i}=\sqrt{GX_{i}^{2}+GY_{i}^{2}+GZ_{i}^{2}}$
ACCma20 [m/s_2_]	Moving average on accelerometer applied on the last 20 samples	$ACCma20_{i}=\frac{1}{20}\sum_{k=i-19}^{k=i}\left(|AX_{k}|+|AY_{k}|+|AZ_{k}|\right)$
ACCdma20 [m/s_2_]	Moving average on accelerometer derivatives applied on the last 20 samples	$\;\begin{array}{cc} & ACCdma20_{i}=\\ & \frac{1}{20T_{s}}\sum_{k=i-19}^{k=i}\left(|\Delta AX_{k}|+|\Delta AY_{k}|+|\Delta AZ_{k}|\right)\\ & \Delta A_{k}=A_{k}-A_{k-1} \end{array}$

**Table 6:** The second benchmark performance comparison of NNs on different inputs.

Group	Model Parameters		Performance (Correlation)			
	**Model and Channels**	**Input Dim**	**X**	**Y**	**Z**	**All**
Best from Table 1	NN on AccGyrMagGRDe	12	0.779	0.728	0.945	0.848
Applicable NN (CFNN2H15)	NN on AccGyrMag	9	0.764	0.771	0.952	0.860
	NN on AccGyrMagIMUf	13	0.776	0.767	0.953	0.862
	NN on AccGyrMagEKFe	12	0.771	0.769	0.952	0.861
	NN on AccGyrMagGRDe	12	0.801	0.783	0.951	0.871
	NN on AccGyrMagEKFeIMUf	16	0.786	0.774	**0.954**	0.867
	**NN on AccGyrMagGRDeIMUf**	16	0.805	**0.805**	0.952	**0.878**
	NN on AccGyrMagEKFeEKFa	15	0.777	0.768	0.952	0.862
	NN on AccGyrMagGRDeGRDa	15	**0.809**	0.798	0.952	0.877
CFNN2H15 with ground truth angles	NN on AccGyrMagANGe	12	0.977	**0.973**	0.960	0.964
	**NN on AccGyrMagANGeIMUf**	16	0.977	0.972	**0.962**	**0.965**

*Bold numbers indicate the best results within each group.*

**Table 7:** Typical 3D position errors after 5, 10, and 15 s of double integrated acceleration estimated by GRDa, EKFa, and CFNN2H34.

	GRDa			EKFa			CFNN2H34a		
**Time**	**X [m]**	**Y [m]**	**Z [m]**	**X [m]**	**Y [m]**	**Z [m]**	**X [m]**	**Y [m]**	**Z [m]**
5 s	7.23	4.97	1.59	4.17	3.75	1.45	**1.51**	**1.15**	**0.61**
10 s	54.69	13.42	6.37	11.22	11.78	5.23	**4.26**	**3.55**	**1.89**
15 s	142.11	35.20	13.73	19.16	18.33	9.44	**10.04**	**7.37**	**4.59**

*Bold values denote that the best performance is obtained by CFNN2H34.*

**Figure A1:**
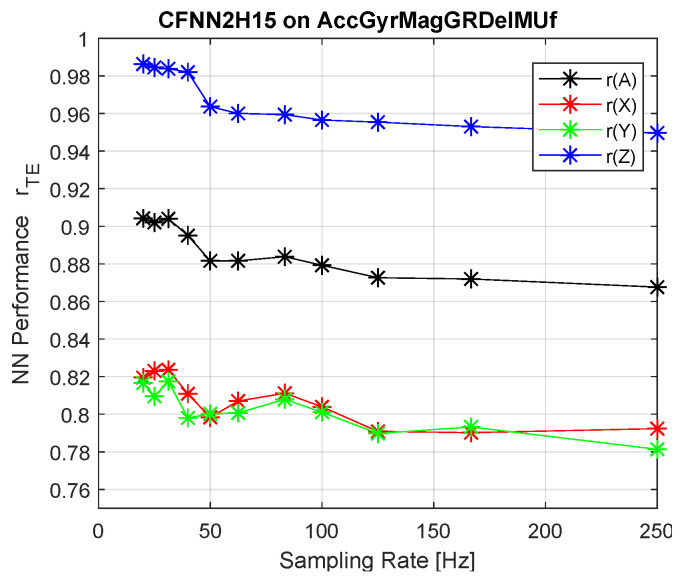
NN performance based on different sampling rates.

**Figure A2:**
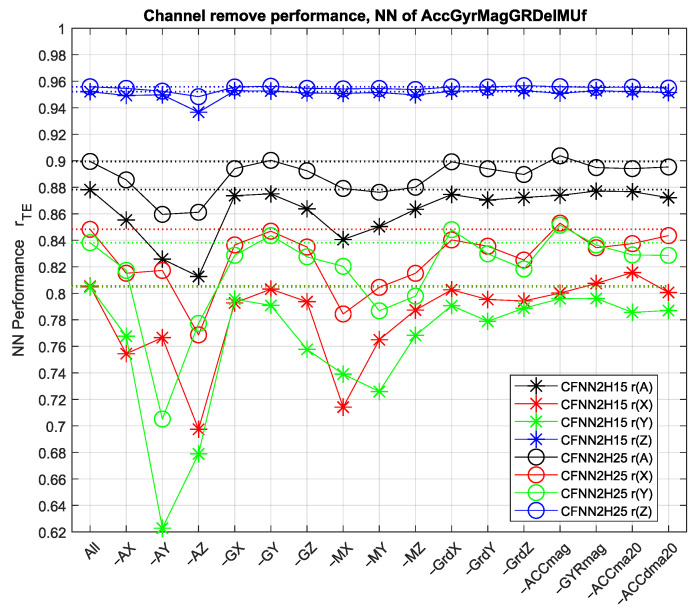
Input channel importance test. The performance drop was compared when skipping each of the input channels. Two NNs are depicted: CFNN2H15 and CFNN2H25.

## Data Availability

The data supporting the findings of this study are available at [50].

## Abbreviations

The following abbreviations are used in this manuscript:

ACC (Acc)	Accelerometer signal
ANGe	Orientation estimation in Euler format
ANGq	Orientation estimation in Quaternion format
CFNN	Cascade-Forward Neural Network (a specific type of shallow network)
CFNN1H18	CFNN with 1 hidden layer and 18 neurons
CFNN2H34	CFNN with 2 hidden layers and 2×34 neurons
CNN	Convolutional Neural Network (not shallow network)
DNN	Deep Neural Network (i.e., not a shallow network)
DOF	Degrees of Freedom
EKF	Extended Kalman Filter
EKFe	EKF-based orientation signal in Euler format
EKFq	EKF-based orientation signal in Quaternion format
FTDNN	Focused Time-Delay Neural Network
GPS	Global Positioning System
GRD	Gradient Descent algorithm
GRDe	GRD-based orientation signal in Euler format
GRDq	GRD-based orientation signal in Quaternion format
GYR (Gyr)	Gyroscope signal
IMU	Inertial Measurement Unit
MAG (Mag)	Magnetometer signal
MARG	Magnetic, Angular Rate, and Gravity
NN	Neural Network
RPY	Roll–Pitch–Yaw signal
SNN	Shallow Neural Network

## Appendix A. Effect of Sampling Rate

In this analysis, the NN performance is examined based on the sampling rate. Our choice of default sampling rate was 100 Hz; however, an analysis was also conducted to better understand the nature of the system. The sampling rates were selected based on integer divisors of the original 1000 Hz, i.e., 50 (1000 Hz/50 = 20 Hz), 40, 32, 25, 20, 16, 12, 10 (default 1000 Hz/10 = 100 Hz), 8, 6, and 4 divisors were evaluated. The best NN was employed; however, a lower number of neurons were applied to collect the results more quickly. The obtained trend is interesting. Figure A1 shows the achieved r(X), r(Y), r(Z), and r(A) performance results based on the input sampling rate. The trend shows a slight performance decrease for the increased sampling rate without any high fluctuations. This outcome was expected, as the lower frequency components can be estimated and the high frequencies are filtered out using lower sampling rate.

The aforementioned analyses suggest that high frequencies are not required for position estimation by double integration based on the estimated accelerometer signals. The appropriate sampling rate can be even lower than the currently used 100 Hz. It is also shown that the estimation accuracy does not degrade at lower sampling rates (e.g., from 100Hz to 50 Hz), and in some cases slightly improves. This is a significant finding for practical implementation. It means that the sampling rate can be reduced to 50 Hz without sacrificing performance, thereby halving the computational load. This is an excellent tradeoff for resource-constrained systems.

## Appendix B. Channel Importance Test

The contribution of each input channel in the NN-based acceleration estimation was tested in a channel skip test. A channel was skipped and the NN was recalculated to check the extent of the performance drop. The performance of the output channels (r(X), r(Y), r(Z)) individually depends on an input channel; therefore, these three output performances were evaluated. For example, based on our observation, the MX input channel influences r(X) output performance but not r(Z). The results are depicted alongside the overall performance, r(A), in Figure A2, which shows the outcome of the channel skip test on two NNs with different numbers of neurons, i.e., 15 (CFNN2H15) and 25 (CFNN2H25). The first point in Figure A2 represents the original performance including all 16 channels according to the previously selected configuration, AccGyrMagGRDeIMUf. The performance drop varies between 0–10% and its average is approximately 1%, representing a relatively small change. This confirms that redundancy exists between the selected 16 channels; moreover, the remaining channels can substitute for the information of the missing channel. In addition, the relative importance of each input channel on each output channel can be observed through the performance metrics:

1 The X output channel highly relies on the AZ, AX, and MX input channels.
2 The Y output channel highly relies on the AY, AZ, and MY input channels.
3 The Z output channel highly relies on the AZ channel.

The biggest drop in overall performance was caused by the absence of the accelerometer or magnetometer channels. As a result, it can be concluded that the NNs rely mostly on the accelerometer and magnetometer channels, as these sensors supply absolute information of the reference vectors.
